# A Proposed New Staff of Outdoor Medical Officers

**Published:** 1920-05-15

**Authors:** 


### THE MINISTRY OF HEALTH.

### A Proposed New Staff of Outdoor Medical Officers.

The National Insurance Acts have already rendered a State medical service a *fait accompli* in respect of a large proportion of the population, so it is only natural and right that the newly formed Ministry of Health, whilst actively engaged in a close study of the great problems of prophylactic medicine and hygiene, should early fasten its attention on the pressing needs of the great public medical service upon which the physical well-being of so many largely depends. Seven years of experience of the National Insurance scheme in actual operation have done much towards a discovery of its limitations and weak points. The war interfered very seriously with the working of the great machine, and much of the work was perforce accomplished in a hand-to-mouth fashion. After making due allowance for these disabilities, however, it is clear that even in the most favourable conditions there are serious weaknesses in the scheme as it exists to-day. One of its most glaring deficiencies is the inadequate nature of its medical service. We speak in respect neither of the number of the panel doctors nor of their work. Our reference is to the complete lack of an official and organised band of consultants and specialists.

### The Insured Person.

The insured person has, in theory, access to any and every possible scientific aid to recovery from his disability, whether the need be of man or appliance. Actually, he may get it, not because the State fulfils its obligations to him, but simply and solely because "Charity never faileth." Of any 100 patients coming under the Insurance Act, at least 50 per cent. would recover from their illnesses quite well without a doctor. The needs of probably another 30-40 per cent. ought to be met adequately by the panel doctor. But what of the remaining 10 to 15 per cent? What official provision is there by the State for these minorities whose illnesses call for expert advice and treatment of a hundred and one different kinds? To what official referee, except in the matter of tuberculosis, can the panel doctor turn in times of doubt and difficulty? Where is the needed extra help to be found? Just where it always has been found—in the voluntary hospitals. The patient does not suffer, perhaps, from the haphazard system, if system it can be called, but the consultant receives not a penny piece for his State service, and the panel doctor, instead of receiving help in the treatment of his serious cases, has them removed altogether from his care, and, with the separation, loses all possibility of increasing his knowledge and skill.

### The State and the Voluntary Spirit.

This condition of things must be remedied, and the relation of the State to the hospital cases amongst those for whom it has accepted liability in respect of medical treatment, be put upon a definite and business-like footing. Meanwhile we must welcome every step in the direction of completeness of organisation and consequent smoothness and perfection of administration. Towards that end the present intention of the Ministry of Health to appoint a staff of whole-time outdoor medical officers of health to act as referees and consultants in connection with the Insurance Acts must be recognised as a serious contribution. The duties of these gentlemen will be partly clinical and partly administrative. The former will include the examination of insured patients referred to them by approved societies or doctors, advising societies and doctors on questions of incapacity for work, and advising doctors on matters of diagnosis and treatment. In cases of special difficulty specialists will be available.

The administrative work will embrace the examination of health insurance medical certificates, records, reports, and prescriptions, and the making of inquiries of insurance practitioners respecting points that may arise in these matters. The medical officers will, further, undertake any other duties required by the Ministry of Health in connection either with insurance medical work or other branches of the work of the Ministry.

### The New Appointments.

For this work in England and Wales twenty-one men are to be appointed, together with a number of part-time officers, at a later date. To commence with, the position will be as follows: For 58,000 odd square miles of country there will be twenty-one medical officers, eighteen for England and three for Wales, or one man to an average of 2,760 square miles. Successful applicants will be medical men of not less than ten years' standing and of extensive experience in general practice in industrial centres. The salary will be £1,000 per annum, with a yearly increase of £50 up to a maximum of £1,400, with no war bonus. Officers appointed to this scale will be Civil Servants, with the usual pension rights. Each man must reside in the area assigned to him. No mention is made of travelling allowances, but it is doubtful whether anything but a pair of seven-league boots would meet the case. The stipulation that the men must reside in the area assigned to them would appear to be superfluous, since it looks as though they will scarcely have time to reside anywhere.

### What They Imply.

A man who has spent at least five years in one of the most expensive trainings for any profession, and probably another two years in gaining practical experience in hospital appointments either for nothing or at most a mere pittance, after ten years' gruelling in a big industrial centre, is to be paid £1,000, and for this he is to undertake multitudinous duties of an administrative nature, in addition to being the sole Insurance Consultant over an area

**The Ministry of Health—(*continued*).**

of 2,760 square miles. What the clinical work alone will involve in time, travelling, expenditure of energy, we do not like to think. In our judgment at least twice the number of men intended should be appointed, with a salary that shall bear some relation to their age, professional attainments, and general status. National Insurance is a vast organisation and no petty measures can result in anything but waste of money, confusion, and disappointment. There is a crying need for the step contemplated, but unless it be undertaken with a full appreciation of the extent of the work to be done, and therefore in an adequate measure, it had better be dropped. But something must be done. This is the age of reconstruction, and unless the partial machinery of the public medical service be complete, at least in respect of each attempted step towards its ultimate perfection, it will be better, in our judgment, to cease all efforts in that direction and to subject the whole scheme of National Insurance to drastic revision and reconstruction.

The Panel Doctor's View.

From the panel doctor's point of view the scheme, adequately conceived and launched, should be of great benefit. It will provide him with what at present simply does not exist—*viz.*, an official referee to whom he can turn in time of doubt and difficulty, knowing that both he and his patient are asking no favour, but are simply drawing upon a reserve to which they have every right. Further, the doctor will suffer no detriment to his own reputation and practice, such as is liable to occur when the second opinion has, perforce, to be represented by a colleague in his immediate neighbourhood. The average patient inevitably regards the second doctor as belonging to a superior order of beings, however circumspect and unassuming his behaviour may be. When such a man is either a recognised private consultant or an official referee the magic word "Specialist" is on everyone's tongue, and no harm results. He comes upon the scene as a star of great magnitude and passes on his meteoric course.

Patients Themselves.

Lastly—how will the patients themselves be affected by the proposed addition to their medical service? Numbers of cases of a more or less doubtful nature, though not of serious character at the time, will more readily be given the advantage of a second opinion with the increased facilities, and thus safeguarded in many instances against future possible grave developments which so often occur in cases imperfectly understood. The sense that they are being treated fairly and justly, that those in authority really have their welfare at heart and make honest endeavours to stand by their promises and to fulfil their obligations, will help towards imbuing the great mass of insured persons with that spirit of contentment born of confidence in those in high places, which is so needed to-day.

